# Supplementary material for: Interferon-α-Induced Dendritic Cells Generated with Human Platelet Lysate Exhibit Elevated Antigen Presenting Ability to Cytotoxic T Lymphocytes
Source: Vaccines (Basel). 2020 Dec 24;9(1):10. doi: 10.3390/vaccines9010010 (PMC7823331; doi:10.3390/vaccines9010010)
Supplement: Supplementary file 1 [file vaccines-09-00010-s001.pdf]

vaccines

MDPI

Table 1. Viability and yield of IFN-DCs in DCO-K only, with human AB serum, and with HPL conditions.

	DCO-K only	+ human AB serum	+ HPL*
Yield (DC/Monocyte)	14.3%	9.7%	25.2%
Viability	85.5%	61.4%	95.5%

* human platelet lysate.

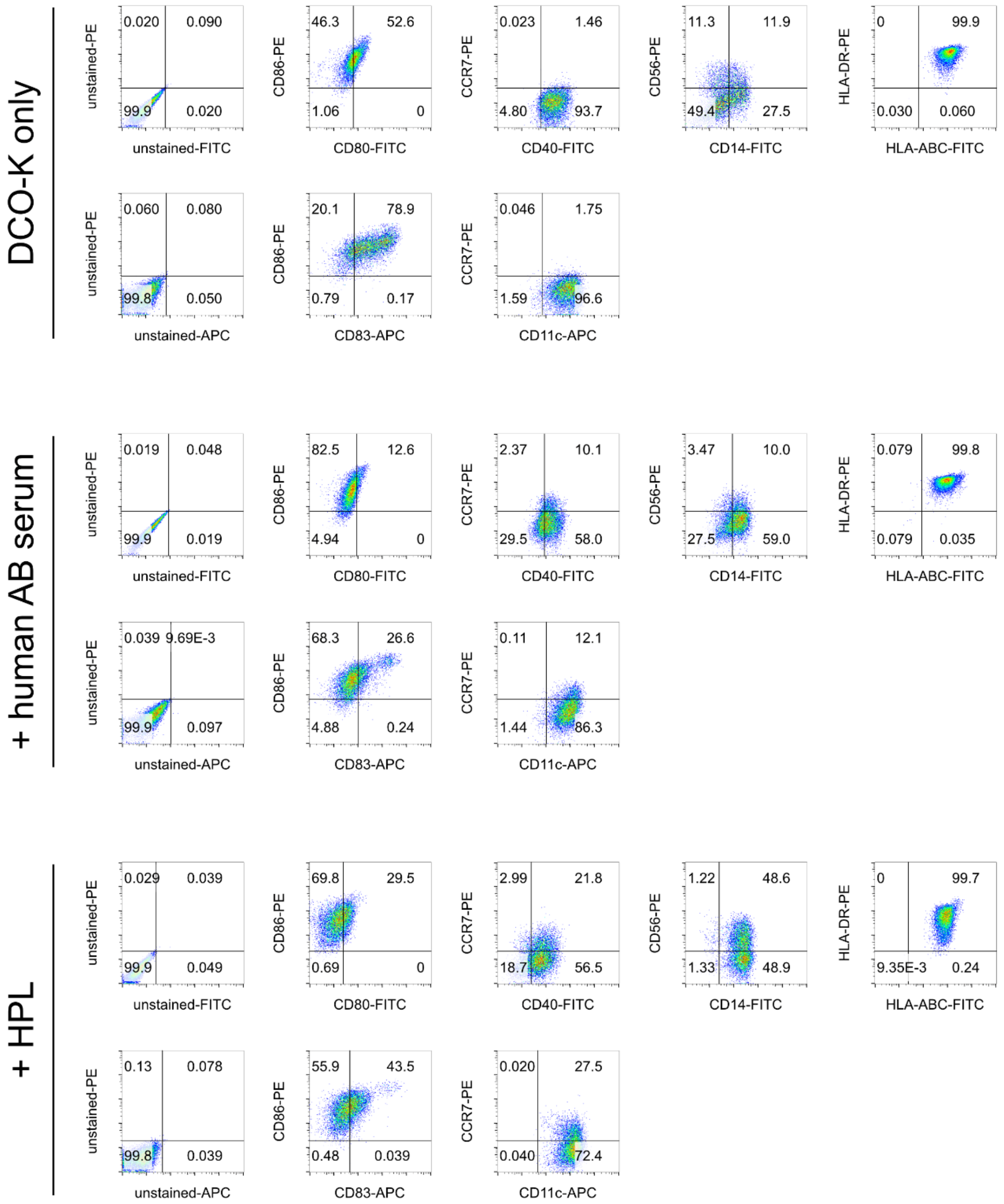

Figure 1. Panels of surface marker expression on IFN-DCs either with human AB serum or with HPL in DCO-K medium. Dot plots of representative IFN-DC-related markers are shown. Quadrant gate

was determined as percentage of isotype controls of more than 99%. The numbers in four squares of the panels indicate percentage of each fraction.

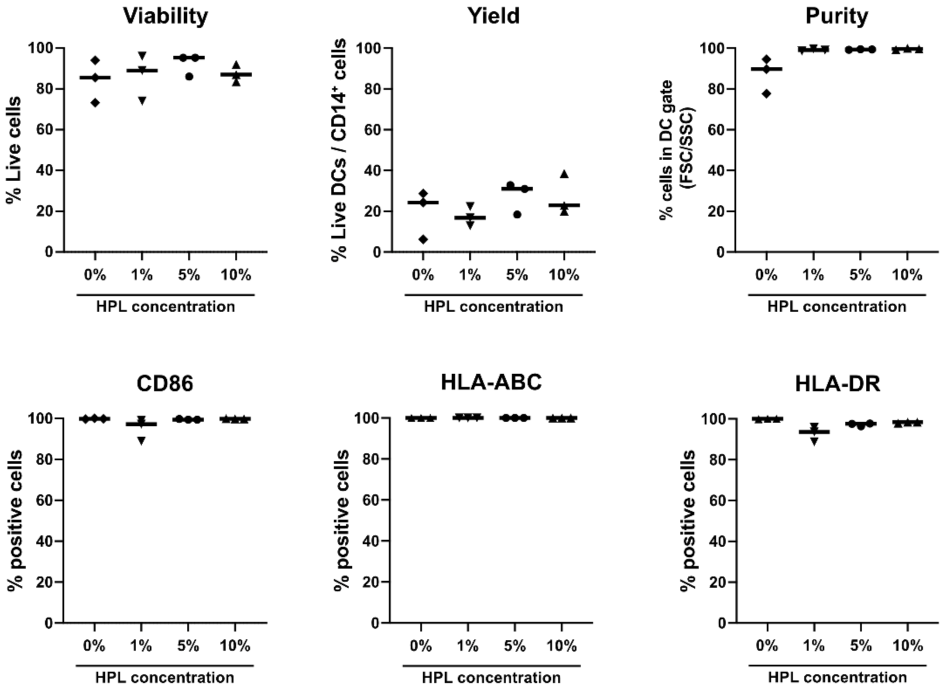

Figure 2. Verification of the optimal concentration of HPL for IFN-DC generation. The cell viability, yield, and purity are shown in the upper panel. The lower panel shows typical markers of DC phenotypes such as CD86, HLA-ABC, and HLA-DR (n = 3).

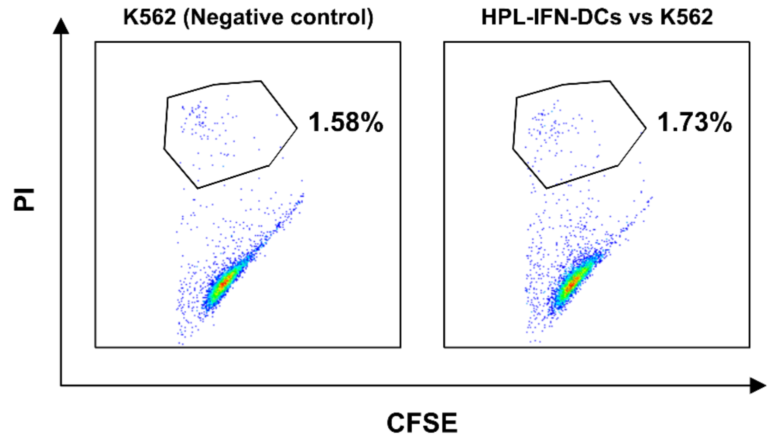

Figure 3. HPL-IFN-DCs have little tumor killing activity. At first, human chronic myeloid leukemia cell line K562 dyed with 5-(and -6)-carboxyfluorescein diacetate succinimidyl ester (CFSE; 5 μM) was prepared. HPL-IFN-DCs were co-cultured with CFSE-labeled K562 at a ratio of E:T = 50:1 for 18 h. Unstained K562 cells were used as a negative control for co-culture instead of HPL-IFN-DCs. Then, the collected cells were stained with propidium iodide (PI) and analyzed by flow cytometry. $CFSE^+/PI^+$ fractions were considered as dead $CFSE^+$ K562 cells killed by effector cells. Compared with the negative control, no direct tumor killing activity was observed in HPL-IFN-DCs.

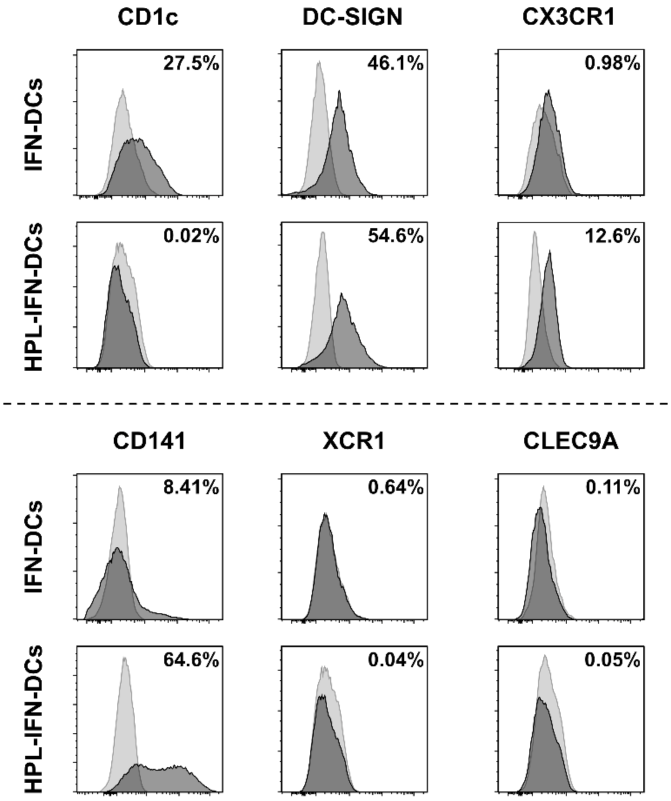

Figure 4. The expressions of cell surface markers associated with dermal DCs. The expressions of the dermal DC markers CD1c, CD141, XCR1, CLEC9A, DC-SIGN, and CX3CR1 were examined in mature IFN-DCs and HPL-IFN-DCs. The percentages in the panels indicate the positive ratio of each marker in live DCs. The dark gray histogram indicates the cells stained by each antibody, and the light gray histogram indicates the non-stained cells.
